# β-Nicotinamide Mononucleotide Enhances Skin Barrier Function and Attenuates UV-B-Induced Photoaging in Mice

**DOI:** 10.3390/antiox14121424

**Published:** 2025-11-27

**Authors:** Sung Jin Kim, Sullim Lee, Yea Jung Choi, Minseo Kang, Junghwan Lee, Gwi Seo Hwang, Seok-Seon Roh, Mu Hyun Jin, Sangki Park, Minji Park, Ho Song Cho, Ki Sung Kang

**Affiliations:** 1 College of Korean Medicine, Gachon University, Seongnam 13120, Republic of Korea; sungjinkim001@gmail.com (S.J.K.); domdada22@gachon.ac.kr (Y.J.C.); seoul@gachon.ac.kr (G.S.H.); 2 Department of Life Science, College of Bio-Nano Technology, Gachon University, Seongnam 13120, Republic of Korea; sullimlee@gachon.ac.kr (S.L.); bana825@gachon.ac.kr (M.K.); 3 School of Biosystems and Biomedical Sciences, Korea University, Seoul 02841, Republic of Korea; texans117099@gmail.com; 4 Science Research Park, LG Household and Healthcare Ltd., Seoul 07795, Republic of Korea; rssdr@hanmail.net (S.-S.R.); mhjin@lghnh.com (M.H.J.); skparkm@lghnh.com (S.P.); minjiipark1@lghnh.com (M.P.)

**Keywords:** UV-B–induced photoaging, nicotinamide mononucleotide (NMN), skin barrier function, oxidative stress, extracellular matrix remodeling

## Abstract

Ultraviolet B (UV-B) radiation significantly contributes to skin photoaging, which is characterized by epidermal thickening, collagen degradation, wrinkle formation, barrier dysfunction, and oxidative stress. Nicotinamide mononucleotide (NMN), a key precursor of nicotinamide adenine dinucleotide, regulates cellular energy metabolism and antioxidant defense and demonstrates anti-aging effects in animal models. Here, we investigated the protective effects of oral NMN supplementation against UV-B-induced photoaging in SKH-1 hairless mice. Over a 10-week experimental period, oral NMN administration significantly alleviated epidermal hypertrophy, reduced wrinkle formation and skin surface roughness, improved hydration and elasticity, and restored transepidermal water loss to near-normal levels. Histological analyses revealed marked preservation of collagen fiber density and attenuation of dermal matrix degradation. Furthermore, NMN supplementation inhibited the phosphorylation of MAPK signaling components (ERK, JNK, and p38), suppressed pro-inflammatory cytokine (TNF-α and IL-6) and matrix-degrading enzyme (MMP-1) expression, and restored hyaluronan synthase (HAS-1 and HAS-2) expression. Additionally, NMN enhanced the systemic antioxidant defense, as indicated by the restored superoxide dismutase activity. Thus, NMN has multi-layered protective effects against UV-B–induced skin aging by modulating oxidative stress, inflammatory signaling, extracellular matrix remodeling, and hyaluronic acid metabolism.

## 1. Introduction

Photoaging, which refers to premature skin aging caused by chronic exposure to ultraviolet (UV) radiation, is characterized by wrinkle formation, loss of skin elasticity, decreased hydration, epidermal thickening, and degradation of dermal extracellular matrix (ECM) components, such as collagen and hyaluronic acid (HA) [1,2,3]. Specifically, UV-B radiation penetrates the epidermis and induces oxidative stress, inflammatory responses, and activation of signaling pathways including mitogen-activated protein kinases (MAPKs), which subsequently upregulate matrix metalloproteinases (MMPs), accelerating ECM degradation [4,5,6]. These processes contribute to visible and functional skin decline, highlighting the need to develop effective preventive and therapeutic strategies against photoaging.

Nicotinamide mononucleotide (NMN), a key precursor of nicotinamide adenine dinucleotide (NAD^+^), is a bioactive compound that demonstrates multiple physiological functions, including enhancing cellular energy metabolism, activating sirtuin pathways, and boosting antioxidant defense systems [7,8,9]. Oxidative stress and inflammation primarily contribute to UV-B-induced skin damage, highlighting the potential of NMN to mitigate photoaging [10,11,12]. Previous studies have shown that NAD^+^ precursors, including NMN, improve skin barrier function, increase hydration, and preserve dermal collagen content [13].

However, most studies investigating NMN’s protective effects against UV-B-induced skin damage have relied on its administration via intraperitoneal (IP) injections. For example, β-NMN delivered via IP injection alleviated oxidative damage and collagen degradation in UV-B–irradiated mice [12], and NMN recruited glutathione (GSH) to enhance GPX4-mediated ferroptosis defense under UV stress [14]. Although several mechanistic studies have evaluated NMN, most have relied on parenteral administration, limiting their translational relevance to humans. In contrast, oral supplementation is safer, more convenient, and more applicable to nutraceutical or cosmeceutical development. However, few studies have examined the protective effects of orally administered NMN against UV-B-induced skin photoaging. Importantly, a recent study demonstrated that oral NMN administration prevents tumor formation in UV-exposed mice, underscoring the need to consider and further investigate this route of delivery [15].

Furthermore, the impact of NMN on specific molecular and functional endpoints, including inflammatory cytokine expression, MMP activity, hyaluronan synthase (HAS-1/2) regulation, systemic antioxidant defense, epidermal thickness, collagen fiber integrity, hydration, elasticity, and wrinkle formation, has not been comprehensively evaluated. Addressing these gaps is essential to establishing the therapeutic potential of NMN in skin aging research.

In this study, we systematically investigated the protective and restorative effects of oral NMN administration on UV-B-induced skin damage in a mouse model by comprehensively assessing skin barrier function, hydration, elasticity, wrinkle formation, histological changes, ECM integrity, MAPK signaling, inflammatory cytokines, MMPs, HAS-1/2 expression, and systemic antioxidant activity. To our knowledge, this is the first study to demonstrate the multifaceted benefits of oral NMN supplementation in UV-B-induced photoaging, providing novel insights into its potential as a preventive or therapeutic intervention.

## 2. Materials and Methods

### 2.1. Reagents

β-Nicotinamide mononucleotide (β-NMN; ≥99% purity, Sigma-Aldrich, St. Louis, MO, USA) and collagen (Sigma-Aldrich, St. Louis, MO, USA) were purchased from EffePharm Co., Ltd. (Shanghai, China). Other reagents and chemicals were of analytical grade.

### 2.2. Animals

SKH-1 hairless mice (7 w, female) (Orient Bio Inc., Seongnam, Gyeonggi-do, Republic of Korea) were acclimated to the animal facility for one week prior to the experiment. Mice were maintained under controlled environmental conditions at a temperature of 20–23 °C, relative humidity of 60%, and a 12 h light/dark cycle. Standard chow diet and water were provided ad libitum. All experimental procedures were approved by the Institutional Animal Care and Use Committee (IACUC) of Gachon University (approval no. GU1-2025-IA0001) and conducted in accordance with its guidelines.

### 2.3. UV-B-Induced Photoaging and Treatment

**Figure 1:**
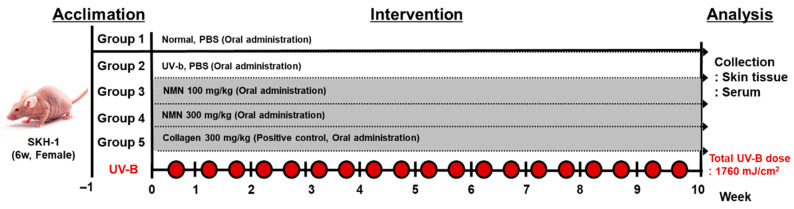
Experimental timeline of NMN treatment in SKH-1 hairless mice exposed to UV-B irradiation. The total cumulative UV-B dose administered over the 10-week period was 1760 mJ/cm^2^.

Mice were randomly assigned to five experimental groups (*n* = 5 per group): vehicle, UV-B, UV-B + NMN 100 mg/kg, UV-B + NMN 300 mg/kg, and UV-B + Collagen 300 mg/kg (positive control). Photoaging was induced using a custom-built UV-B lamp, and irradiance was measured using a UV radiometer (RMX-3 W, Vilber Lourmat, Collégien, France). UV-B irradiation was administered three times per week for 10 weeks, starting at 50 mJ/cm^2^ and increasing by 10 mJ/cm^2^ every two exposures, up to a final dose of 140 mJ/cm^2^ [16] (Figure 1). The total cumulative UV-B dose administered over the 10-week period was 1760 mJ/cm^2^. NMN or collagen was dissolved in PBS and orally administered once daily throughout the 10-week UV-B exposure period.

### 2.4. Skin Moisture Measurement

Skin moisture was assessed using an AramoSG^®^ ASG 200F (Aram Huvis Co., Ltd., Seongnam, Republic of Korea). The probe was gently applied to the dorsal skin of each mouse, and three consecutive measurements were recorded for each animal and averaged for analysis.

### 2.5. Skin Elasticity Measurement

Skin elasticity was assessed using a Ballistometer BLS785 (Dia-Stron Ltd., Andover, UK). The instrument applied a standardized force to the dorsal skin surface, and the resulting indentation and rebound were noted. The measurements were repeated three times for each animal, and the average value was used for analysis.

### 2.6. Transepidermal Water Loss (TEWL) Measurement

TEWL was assessed using GPSkinBarrier^®^ (GPOWER Inc., Seoul, Republic of Korea). The sensor was applied to the dorsal skin, and measurements were performed in triplicates. TEWL was determined by measuring the amount of water evaporating from the skin surface, which serves as an indicator of skin barrier integrity [17].

### 2.7. Skin Wrinkle and Structure Assessment

Skin wrinkle formation and structure were assessed using the Visioline^®^ VL650 Replica kit (Courage+Khazaka electronic GmbH, Cologne, Germany). Silicone replicas of the dorsal skin were prepared to capture the surface topography, which was subsequently analyzed using a Visioline^®^ VL650 system. The wrinkle parameters, including depth (µm), length (mm), and area (mm^2^), were quantified.

### 2.8. Western Blot Analysis

Proteins were extracted from mouse dorsal skin tissues using RIPA lysis buffer (Cell Signaling Technology, Danvers, MA, USA) supplemented with a protease inhibitor cocktail and 1 mM phenylmethylsulfonyl fluoride (PMSF). Equal amounts of protein were separated by SDS-PAGE and transferred onto PVDF membranes. The membranes were incubated with primary antibodies against ERK1/2 (#9102), phospho-ERK1/2 (Thr202/Tyr204, #9101), JNK (#9252), phospho-JNK (Thr183/Tyr185, #9251), p38 (#9212), and phospho-p38 (Thr180/Tyr182, #9211) (all from Cell Signaling Technology), followed by incubation with HRP-conjugated secondary antibody (anti-rabbit IgG, #7074; Cell Signaling Technology). Protein signals were visualized using ECL Advance Western blotting Detection Reagents (GE Healthcare, Buckinghamshire, UK) and imaged using the LAS 4000 system (Fujifilm, Tokyo, Japan). Protein band quantification was performed using image analysis software [18].

### 2.9. Reverse Transcription Quantitative Polymerase Chain Reaction (RT-qPCR) Analysis

Total RNA was isolated from mouse dorsal skin tissues using the RNeasy Mini Kit (Qiagen, Hilden, Nordrhein-Westfalen, Germany) following the manufacturer’s instructions. RNA purity and concentration were assessed using NanoDrop spectrophotometry (Thermo Fisher Scientific, Waltham, MA, USA). cDNA was synthesized from 1 μg of total RNA using a cDNA Reverse Transcription Kit (Thermo Fisher Scientific) under the following conditions: 25 °C for 10 min, 37 °C for 120 min, and 85 °C for 5 min. The mRNA expression levels of TNF-α, IL-6, MMP-1, HAS-1, HAS-2, and GAPDH were determined using sequence-specific primers (Bioneer, Daejeon, Republic of Korea; Table 1). PCR amplification was performed on a QuantStudio™ 3 Real-Time PCR System (Thermo Fisher Scientific) under the following cycling conditions: initial denaturation at 95 °C for 10 min, followed by 40 cycles of 95 °C for 15 s and 60 °C for 1 min. Relative gene expression was calculated using GAPDH as the internal control and the ΔΔCt method [19]. All reactions were performed in triplicate.

### 2.10. Superoxide Dismutase (SOD) Activity Assay

Serum samples were collected from mice and analyzed for SOD activity using a Superoxide Dismutase activity assay kit (Cat. No. ab65354, Abcam, Cambridge, UK) according to the manufacturer’s instructions. Briefly, SOD activity was determined based on its ability to scavenge superoxide radicals, and absorbance was recorded at 450 nm to quantify the enzymatic activity [20].

### 2.11. Histological Staining and Analysis

Skin tissues were harvested post-mortem, fixed in 10% neutral-buffered formalin for 24 h, and embedded in paraffin. The sections were stained with Hematoxylin & Eosin (H&E) and Masson’s trichrome. The stained sections were examined and imaged using a light microscope. Epidermal thickness and collagen fiber density were quantified using ImageJ 1.52 software (National Institutes of Health, Bethesda, MD, USA) [21].

### 2.12. Statistical Analysis

All data are presented as mean ± standard deviation (SD). Comparisons between groups were performed using one-way analysis of variance (ANOVA) followed by Tukey’s post hoc test when significant differences were detected using GraphPad Prism 9.0 (GraphPad Software, San Diego, CA, USA). Statistical significance was set at a 95% confidence level (*p* < 0.05). Significant differences are indicated in the figures by asterisks (*) or hash symbols (#).

## 3. Results

### 3.1. Effect of NMN Administration on Body Weight in UV-B–Exposed Mice

Body weight was monitored throughout the 10-week experimental period, during which all the groups exhibited a gradual increase. No statistically significant differences were detected among the groups, indicating that oral administration of NMN or collagen did not exert a measurable impact on body weight trajectories in UV-B–exposed mice (Supplementary Table S1).

### 3.2. Effects of NMN Supplementation on Skin Barrier Function

**Figure 2:**
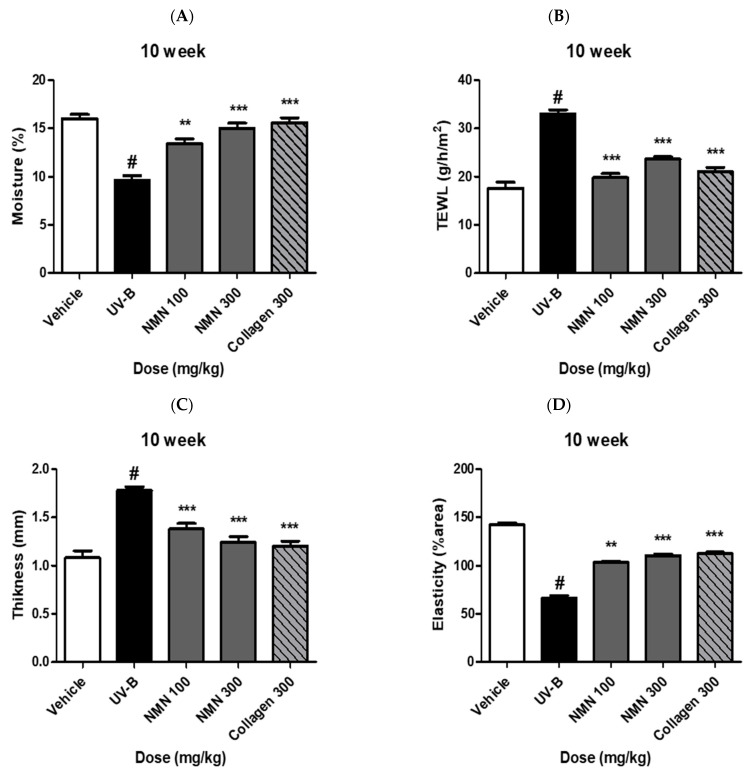
Protective effects of NMN against UV-B-induced skin damage in SKH-1 hairless mice. (**A**) Skin thickness, (**B**) skin hydration, (**C**) transepidermal water loss (TEWL), and (**D**) skin elasticity were measured at baseline (week 0) and after 10 weeks of UV-B irradiation with or without NMN. Data are expressed as mean ± standard deviation (SD). Statistical significance: # *p* < 0.001 vs. vehicle group; ** *p* < 0.01, *** *p* < 0.001 vs. UV-B group.

Skin hydration did not differ significantly among the groups at baseline. After ten weeks, the UV-B group showed a marked reduction in skin hydration compared to the vehicle group, with a decrease of 7.8 ± 0.9%. NMN supplementation improved hydration in a dose-dependent manner, with decreases of 4.6 ± 0.6% in the NMN100 group, 3.4 ± 0.5% in the NMN300 group, and 2.6 ± 0.4% in the Coll300 group, indicating partial recovery compared with the UV-B group (Figure 2A).

TEWL levels were significantly elevated in the UV-B group compared to the vehicle group at week 10, increasing by 18.8 ± 1.6 g/h/m^2^. All NMN-treated groups exhibited a significant reduction in TEWL, with increases of 5.4 ± 0.7 g/h/m^2^ in the NMN100 group, 9.2 ± 1.0 g/h/m^2^ in the NMN300 group, and 8.0 ± 0.9 g/h/m^2^ in the Coll300 group, demonstrating dose-dependent improvement and partial restoration of the barrier function (Figure 2B).

Skin thickness was comparable among the groups at baseline. Following ten weeks of UV-B irradiation, skin thickness increased by 0.91 ± 0.08 mm in the UV-B group compared to the vehicle group. Both NMN-treated groups and the collagen-positive control group showed a significant reduction in epidermal hypertrophy, with increases of 0.55 ± 0.05 mm in the NMN100 group, 0.43 ± 0.04 mm in the NMN300 group, and 0.31 ± 0.04 mm in the Coll300 group, suggesting that NMN supplementation attenuated UVB-induced epidermal thickening (Figure 2C).

Skin elasticity, measured using a Cutometer, was similar among the groups at baseline. By week 10, UV-B exposure significantly reduced elasticity, which was partially restored by NMN administration in a dose-dependent manner: 21.0 ± 2.1% area for NMN100, 10.8 ± 1.8% area for NMN300, and 8.4 ± 1.2% area for Coll300, indicating recovery of the mechanical properties impaired by UV-B irradiation (Figure 2D).

### 3.3. Effects of NMN Administration on Skin Wrinkles and Roughness

**Figure 3:**
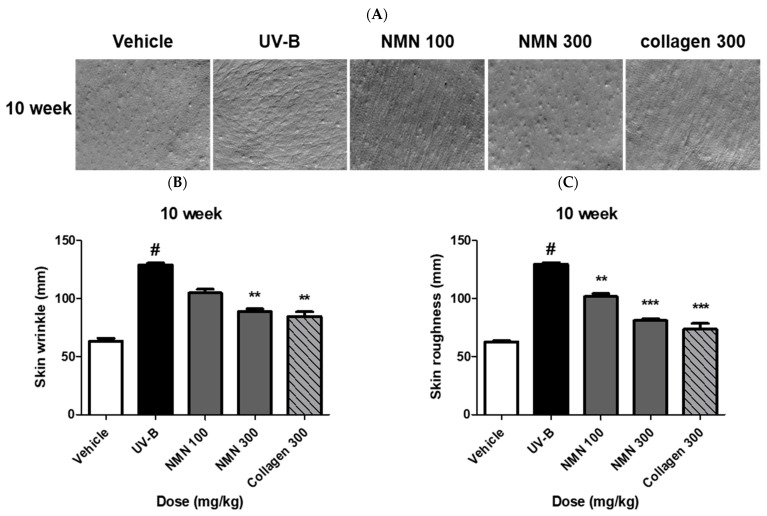
Effects of NMN administration on wrinkle formation and skin roughness in UV-B–irradiated SKH-1 hairless mice. Representative morphological and quantitative analyses of skin wrinkles and roughness were performed using a Visioline^®^ VL650 replica system. (**A**) Representative skin replica images of the experimental groups. (**B**) Quantitative analysis of wrinkle depth and severity. (**C**) Quantitative evaluation of skin surface roughness. Data are expressed as mean ± standard deviation (SD). Statistical significance: # *p* < 0.001 vs. vehicle group; ** *p* < 0.01, and *** *p* < 0.001 vs. UV-B group.

To evaluate the effects of NMN on wrinkle formation, we analyzed silicone replicas of the dorsal skin (Figure 3A). At baseline, the wrinkle depths were similar among the groups. By week 10, the UV-B group showed a pronounced increase from 61.46 ± 1.9 mm at baseline to 129.02 ± 3.2 mm, whereas the vehicle group remained nearly unchanged, increasing from 63.00 ± 3.3 mm to 63.36 ± 5.2 mm. NMN supplementation significantly attenuated this increase in a dose-dependent manner, with values rising from 60.57 ± 2.5 mm to 104.69 ± 7.3 mm in the NMN100 group, from 61.32 ± 1.7 mm to 88.80 ± 4.7 mm in the NMN300 group, and from 59.67 ± 1.7 mm to 84.44 ± 9.0 mm in the Coll300 group, indicating a substantial improvement compared to that in the UV-B group (Figure 3B).

Skin surface roughness was also evaluated at week 10. The UV-B group exhibited a marked increase from 61.15 ± 2.2 mm at baseline to 129.57 ± 2.6 mm, whereas the vehicle group remained nearly unchanged, increasing slightly from 62.40 ± 1.9 mm to 62.58 ± 2.3 mm. Significant improvements were observed in the NMN-treated and collagen positive control groups, with roughness increasing from 60.10 ± 2.0 mm to 101.50 ± 5.9 mm in the NMN100 group, from 59.19 ± 3.6 mm to 80.98 ± 3.2 mm in the NMN300 group, and from 59.01 ± 1.8 mm to 73.39 ± 11.2 mm in the Coll300 group, demonstrating effective attenuation of UV-B-induced roughness (Figure 3C).

### 3.4. Effects of NMN Administration on Epidermal Thickness and Collagen Fibers

**Figure 4:**
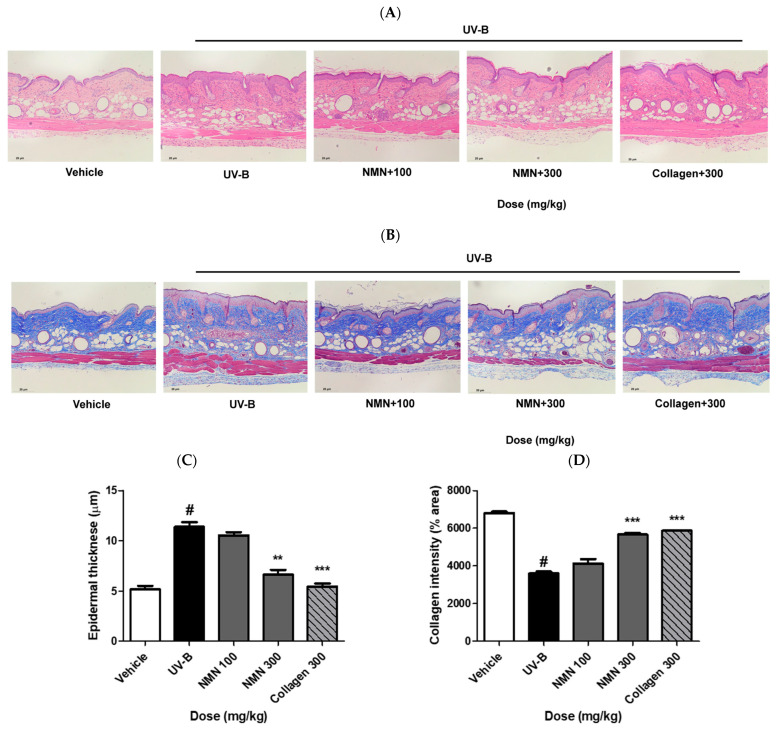
Effects of NMN administration on dermal thickness and collagen fiber density in UV-B–irradiated SKH-1 mice. Dorsal skin tissue was collected 10 weeks after oral NMN administration. (**A**) Representative images of hematoxylin and eosin (H&E) staining for dermal thickness and (**B**) Masson’s trichrome staining for collagen fibers. (**C**) Quantitative analysis of dermal thickness. (**D**) Quantitative analysis of collagen fiber density was performed using ImageJ software. Data are presented as the mean ± standard deviation (SD). Statistical significance: # *p* < 0.001 vs. vehicle group; ** *p* < 0.01, *** *p* < 0.001 vs. UV-B group.

Epidermal thickness and collagen fibers were evaluated using hematoxylin and eosin (H&E) and Masson’s trichrome staining. UV-B irradiation increased epidermal thickness by 6.2 ± 1.3 μm compared to that in the vehicle group. NMN administration markedly attenuated UVB-induced hypertrophy, with thickness decreasing by 0.9 ± 0.8 μm in the NMN100 group, 4.8 ± 1.3 μm in the NMN300 group, and 6.0 ± 1.3 μm in the Coll300 group, approaching the level of the vehicle group. These results indicate a significant reduction in epidermal thickness relative to the UV-B group, with the NMN300 group showing a recovery trend similar to that of the positive control (Figure 4C).

Collagen fiber density, assessed by Masson’s trichrome staining, decreased by 3214.8 ± 298.0% area in the UV-B group compared to that in the vehicle group. Oral NMN supplementation restored collagen density in a dose-dependent manner, with increases of 537.4 ± 571.0% area in the NMN100 group, 2085.2 ± 267.0% area in the NMN300 group, and 2281.8 ± 247.0% area in the Coll300 group, approaching the level of the vehicle group. These results demonstrated a significant recovery of collagen fibers after NMN administration in the UVB-exposed group (Figure 4D).

### 3.5. Effects of NMN Administration on MAPKs Phosphorylation

**Figure 5:**
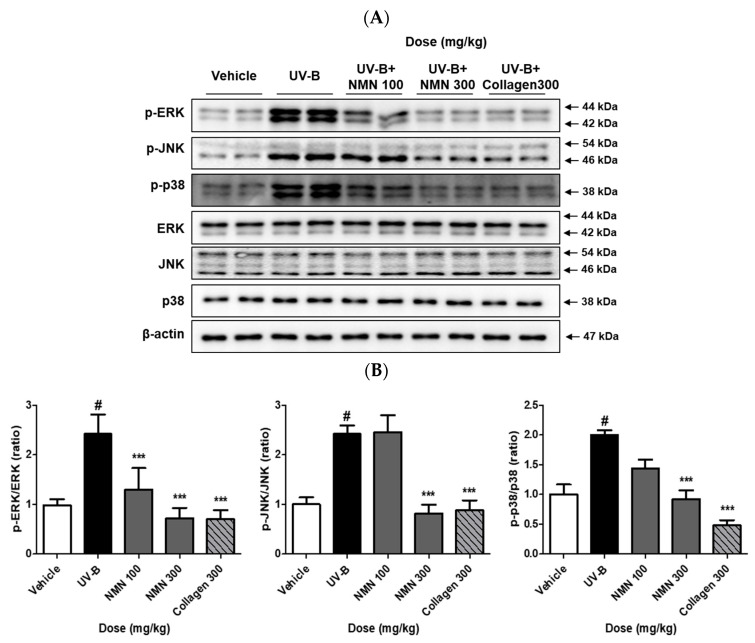
Effects of NMN administration on MAPK phosphorylation in UV-B–irradiated SKH-1 mice. (**A**) Representative Western blot images showing the phosphorylated and total protein levels of ERK, JNK, and p38 in dorsal skin tissues. (**B**) Quantitative analysis of ERK, JNK, and p38 phosphorylation levels. Data are presented as the mean ± standard deviation (SD). Statistical significance: # *p* < 0.001 vs. vehicle group; *** *p* < 0.001 vs. UV-B group.

To assess the effects of NMN on MAPK signaling, we evaluated the phosphorylation levels of ERK, JNK, and p38 by immunoblotting. In the UV-B group, ERK phosphorylation was significantly increased compared to that in the vehicle group (1.0 ± 0.3 vs. 2.4 ± 0.9). Both NMN100 and NMN300 significantly suppressed ERK phosphorylation in a dose-dependent manner, decreasing to 1.3 ± 0.9 in the NMN100 group and to 0.7 ± 0.5 in the NMN300 group. Coll300 showed a similar inhibitory effect, with ERK phosphorylation at 0.7 ± 0.4, indicating comparable inhibition by NMN300 to that of the positive control (Figure 5B).

UV-B irradiation markedly elevated JNK phosphorylation compared to the vehicle group (1.0 ± 0.3 to 2.4 ± 0.4). NMN300 and Coll300 significantly reduced JNK phosphorylation to 0.8 ± 0.4 and 0.9 ± 0.5, respectively. NMN100 had a minimal effect, with JNK phosphorylation at 2.5 ± 0.7 (Figure 5B).

Similarly, p38 phosphorylation significantly increased in the UV-B group compared to that in the vehicle group (1.0 ± 0.4 vs. 2.0 ± 0.2). NMN supplementation reduced p38 phosphorylation in a dose-dependent manner, to 1.4 ± 0.3 in the NMN100 group, 0.9 ± 0.3 in the NMN300 group, and 0.5 ± 0.2 in the Coll300 group, demonstrating effective inhibition by NMN comparable to that of the positive control (Figure 5B).

### 3.6. Effects of NMN Administration on mRNA Expression of Genes Related to Inflammation, Wrinkle Formation, and Skin Hydration

**Figure 6:**
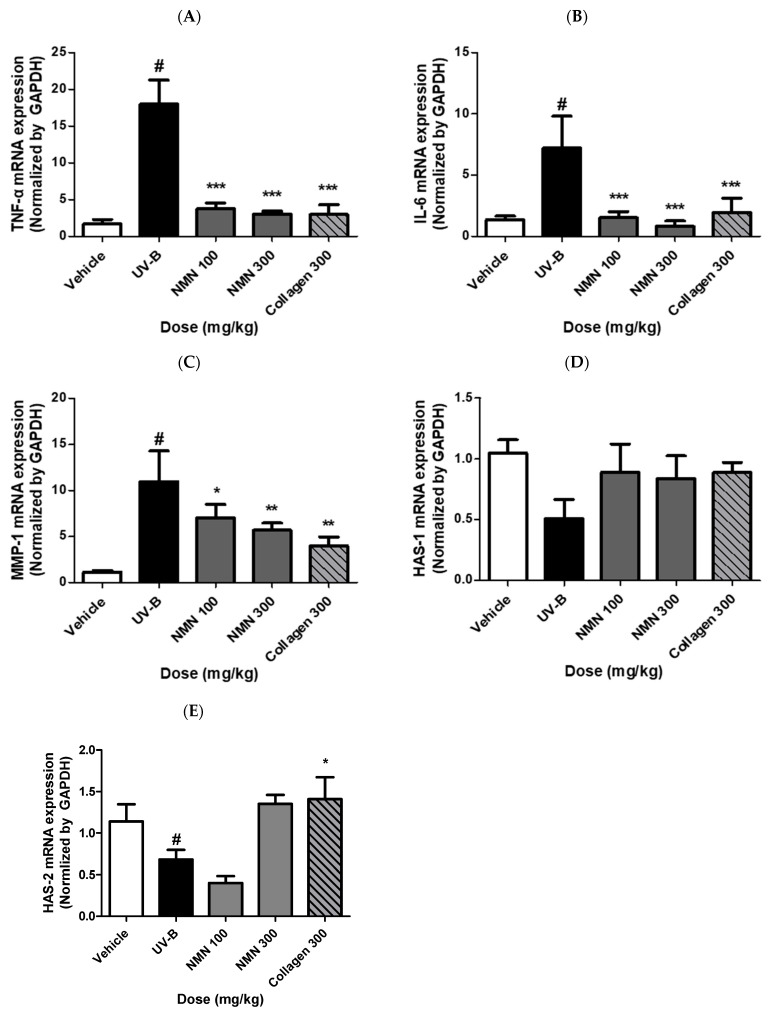
Effects of NMN administration on mRNA expression of inflammatory cytokines, MMP-1, and hyaluronan synthases in UV-B–irradiated SKH-1 mice. After 10 weeks of oral NMN administration, dorsal skin tissues were collected and analyzed by RT-qPCR. (**A**,**B**) TNF-α and IL-6, (**C**) matrix metalloproteinase-1 (MMP-1), and (**D**,**E**) hyaluronan synthase-1 (HAS-1) and hyaluronan synthase-2 (HAS-2). Data are expressed as mean ± standard deviation (SD). Statistical significance: # *p* < 0.001 vs. vehicle group; * *p* < 0.05, ** *p* < 0.01, *** *p* < 0.001 vs. UV-B group.

To elucidate the molecular mechanisms underlying the protective effects of NMN, we evaluated the mRNA expression levels of the inflammatory cytokines TNF-α and IL-6, the wrinkle-associated protein MMP-1, and the hyaluronan synthases HAS-1 and HAS-2 using RT-qPCR (Figure 6). UV-B irradiation markedly elevated TNF-α expression relative to that in the vehicle group, increasing to 17.50 ± 7.83-fold. NMN supplementation significantly suppressed TNF-α expression in a dose-dependent manner, decreasing to 2.58 ± 1.25 in NMN100, 2.67 ± 0.67 in NMN300, and 1.17 ± 0.83 in Coll300. The high-dose NMN group showed expression levels comparable to those of the positive control (Figure 6A). Similarly, IL-6 expression increased to 9.36 ± 8.73-fold in the UV-B group compared that to in the vehicle group. NMN supplementation reduced IL-6 expression to 1.27 ± 1.73 in NMN100, 0.55 ± 0.64 in NMN300, and 2.09 ± 4.27 in Coll300 groups (Figure 6B). MMP-1 expression was significantly upregulated by UV-B exposure, reaching 10.78 ± 7.89-fold relative to that of the vehicle. NMN treatment markedly reduced MMP-1 expression, which decreased to 8.89 ± 5.67 in the NMN100 group, 6.22 ± 4.00 in the NMN300 group, and 4.11 ± 2.44 in the collagen-treated positive control group. High-dose NMN exhibited an inhibitory effect comparable to that of the positive control (Figure 6C). HAS-1 expression decreased to 0.46 ± 0.55-fold in the UV-B group compared to the vehicle group. NMN supplementation restored HAS-1 levels to 0.82 ± 1.00 in the NMN100 group, 0.91 ± 0.55 in the NMN300 group, and 0.82 ± 0.18 in the Coll300 group, respectively. The high-dose NMN group showed a significant recovery in HAS-1 expression (Figure 6D). HAS-2 expression was also downregulated by UV-B irradiation, decreasing to 0.67 ± 0.11-fold. NMN treatment enhanced HAS-2 expression to 0.33 ± 0.11 in NMN100, 1.56 ± 0.44 in NMN300, and 1.56 ± 0.89 in the positive control group (Figure 6E). Notably, the high-dose NMN and collagen groups showed a marked upregulation compared to the UV-B group.

Collectively, these findings demonstrate that NMN supplementation attenuates UV-B-induced skin damage by suppressing inflammatory cytokines and MMP-1, while restoring HAS-1 and HAS-2 expression, thereby contributing to improved skin hydration and dermal integrity.

### 3.7. Effects of NMN Administration on SOD Activity in Serum

**Figure 7:**
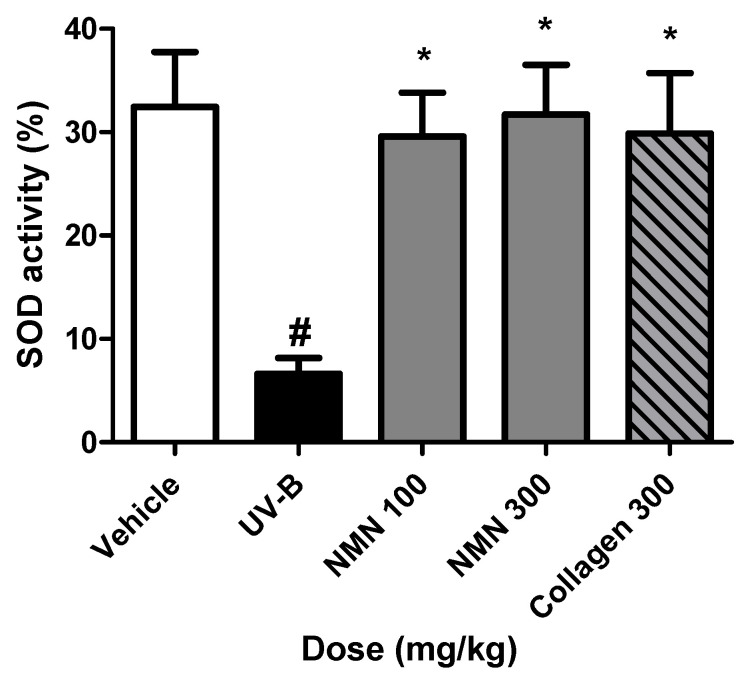
Effects of NMN administration on SOD activity in mouse serum. Mice were orally administered NMN for 10 weeks and exposed to UV-B irradiation to induce oxidative stress. Serum superoxide dismutase (SOD) activity was measured using a WST-based colorimetric assay. Data are expressed as mean ± standard deviation (SD). Statistical significance: # *p* < 0.001 vs. vehicle group; * *p* < 0.05, vs. UV-B group.

To assess the effects of NMN on antioxidant defense, SOD activity in mouse serum was measured after 10 weeks of oral NMN administration (Figure 7). UV-B irradiation significantly decreased SOD activity compared to the vehicle group, from 32.5 ± 10.6% to 6.6 ± 3.0%, corresponding to a decrease of 25.9 ± 10.9%. In contrast, NMN supplementation significantly increased SOD activity in a dose-dependent manner, with a recovery of 23.0 ± 9.0% in the NMN100 group, 25.1 ± 10.0% in the NMN300 group, and 23.3 ± 12.0% in the Coll300 group relative to the UV-B group, indicating a substantial recovery from the UV-B-induced decrease.

## 4. Discussion

In this study, we investigated the protective effects of oral NMN administration on UV-B-induced skin damage and photoaging in SKH-1 hairless mice. Our results demonstrated that NMN significantly attenuated epidermal thickening, improved skin hydration and elasticity, reduced wrinkle formation, preserved dermal collagen density, and enhanced antioxidant defense. At the molecular level, NMN suppressed MAPK signaling, downregulated proinflammatory cytokine and MMP expression, and restored hyaluronan synthase HAS-1 and HAS-2 expression, collectively supporting the maintenance of skin barrier integrity and ECM homeostasis.

UV-B irradiation significantly contributes to photoaging, leading to structural and functional alterations, such as epidermal thickening, collagen degradation, and impaired skin barrier function. In our study, UV-B exposure markedly increased skin thickness, elevated TEWL, and reduced hydration, reflecting barrier disruption. NMN administration significantly alleviated these effects, highlighting its ability to restore barrier function. NMN-induced upregulation of HAS-1 and HAS-2 likely contributes to enhanced epidermal hyaluronic acid synthesis, thereby improving hydration and elasticity. These results are consistent with previous findings showing that NAD^+^ precursors enhance keratinocyte barrier recovery and ECM remodeling [22].

Wrinkles and skin roughness are critical clinical features of photoaging. Our results revealed that NMN treatment, particularly at high doses, significantly reduced wrinkle depth and surface roughness compared to UV-B–exposed controls. Mechanistically, this effect was associated with the suppression of MAPK/AP-1 signaling, which regulates MMP and elastase activity. NMN reduced MMP-1 expression, supporting this mechanism and suggesting the preservation of dermal collagen architecture. Similar findings were reported by Li et al. [23], who demonstrated that NAD^+^ precursors suppress MAPK/AP-1 signaling and prevent collagen degradation in UV-irradiated skin. Taken together, these results highlight the convergence of inflammatory and proteolytic pathways as the central mechanism of NMN-mediated photoprotection.

Histological analyses further supported the protective role of NMN. H&E staining revealed that UV-B-induced epidermal hypertrophy was markedly reduced by NMN, whereas Masson’s trichrome staining demonstrated a dose-dependent restoration of collagen fiber density. These structural improvements likely result from the combined effects of reduced inflammatory signaling, enhanced antioxidant defense, and ECM component stabilization. Previous work by Jung et al. [24] showed that NMN improves mitochondrial function and reduces ROS accumulation in dermal fibroblasts, suggesting that mitochondrial homeostasis may serve as a key mechanistic link between oxidative stress regulation and ECM preservation in the skin.

In addition to structural protection, NMN also modulated molecular signaling pathways. UV-B exposure significantly increased phosphorylation of ERK, JNK, and p38 MAPKs, while NMN supplementation suppressed these responses. This was accompanied by reduced expression of the proinflammatory cytokines TNF-α and IL-6, together with recovery of HAS-1 and HAS-2 expression. These findings suggest that NMN mitigates UV-B-induced inflammatory signaling and promotes skin hydration by restoring hyaluronic acid synthesis, thereby improving both barrier function and mechanical properties [25,26].

Oxidative stress is a critical driver of photoaging, and SOD activity is an important antioxidant defense marker. In this study, UV-B irradiation significantly reduced serum SOD activity, whereas NMN supplementation restored its activity in a dose-dependent manner, which likely contributed to ECM preservation and reduced oxidative damage. Moreover, recent mechanistic studies have indicated that NMN not only increases enzymatic antioxidants but also supports mitochondrial NAD^+^ metabolism and ROS detoxification [27,28,29]. The synergy between mitochondrial homeostasis and enzymatic defense may underlie the broad-spectrum protective effects observed in this study.

Despite these promising findings, several limitations of this study should be noted. First, this study employed a mouse dorsal skin UV-B exposure model, which may not fully recapitulate human skin physiology or the chronic cumulative nature of photoaging. Second, our mechanistic analysis was limited to selected pathways, including MAPK signaling, MMPs, HAS, and SOD. Other pathways, such as SIRT1/3 activation, NAD^+^/NADH ratio modulation, mitochondrial biogenesis, and DNA damage repair, were not addressed and warrant further investigation. Third, only oral NMN administration was evaluated, whereas the potential effects of topical delivery, combined treatments, and long-term supplementation remain unexplored [15,30,31]. Therefore, future research should expand to human skin mimicking models, long-term exposure paradigms, and ultimately, clinical trials to validate NMN’s efficacy in preventing and treating photoaging. Comparative studies on oral versus topical administration, as well as synergistic effects with other antioxidants, may provide deeper insights into its therapeutic potential. In addition, systems-level approaches, such as transcriptomics and metabolomics, could further elucidate the global impact of NMN on skin biology.

In the present study, collagen was used as a positive control to evaluate ECM preservation and the structural outcomes. However, we acknowledge that well-characterized antioxidants, such as NAC or vitamin C, would provide a more direct mechanistic comparison for oxidative-stress–related effects. Incorporating such antioxidant controls in future studies will help delineate the specific contribution of NMN to ROS modulation and further clarify its role within redox-regulated signaling pathways [32]. Although our findings demonstrate concurrent suppression of MAPK signaling and upregulation of HAS-1/HAS-2, we acknowledge that this in vivo study does not establish a direct causal relationship between these events. Future mechanistic experiments, such as the use of selective MAPK inhibitors or rescue approaches in keratinocyte and fibroblast models, will be essential to verify this proposed link [33]. Although NAD^+^ levels, sirtuin activity, and mitochondrial function were not directly assessed in this study, previous research has consistently demonstrated that NMN supplementation enhances NAD^+^ metabolism, activates SIRT1/3, and supports mitochondrial homeostasis, which may contribute to ECM preservation and antioxidant defense. These pathways will be systematically examined in future mechanistic studies to clarify the molecular basis of NMN-mediated protection [26].

Although NMN and collagen showed similar protective effects in our UV-B model, NMN possesses additional mechanistic advantages beyond the structural support provided by collagen alone. NMN not only reduces oxidative stress but also enhances mitochondrial proline biosynthesis and SIRT3-dependent activity, contributing directly to collagen neogenesis. Furthermore, NMN promotes redox balance and mitochondrial resilience, effects not achievable through collagen supplementation alone, suggesting that NMN acts as a more comprehensive anti-aging modulator rather than merely a structural ingredient.

In conclusion, NMN supplementation demonstrated robust protective effects against UV-B-induced skin damage by restoring barrier function, attenuating inflammatory signaling, preserving ECM integrity, and enhancing antioxidant defense. Given its multifunctional role in maintaining skin homeostasis, NMN may be a potential candidate functional food ingredient or cosmeceutical component for further investigation in the context of skin health and photoaging prevention [22,34].

## 5. Conclusions

In this study, we demonstrated that oral administration of NMN exerts significant protective effects against UV-B-induced skin damage and photoaging in SKH-1 hairless mice. NMN supplementation improved multiple skin health parameters, including barrier function, hydration, elasticity, wrinkle formation, and dermal collagen preservation. Histological analyses confirmed the attenuation of epidermal thickening and restoration of collagen fiber density, indicating structural protection at the tissue level.

At the molecular level, NMN inhibited MAPK phosphorylation, reduced the expression of proinflammatory cytokines TNF-α and IL-6 and wrinkle-associated proteins (MMP-1), and restored the expression of hyaluronan synthases HAS-1 and HAS-2. These findings suggest that NMN not only suppresses UV-B-induced inflammatory and proteolytic pathways but also promotes hyaluronic acid synthesis, contributing to improved hydration and elasticity. Furthermore, NMN enhanced the systemic antioxidant defense by restoring serum SOD activity, highlighting its role in counteracting oxidative stress.

Taken together, these findings suggest that NMN mitigates multiple hallmarks of photoaging, including barrier dysfunction, ECM degradation, inflammation, and oxidative stress, through the coordinated regulation of structural, biochemical, and molecular pathways. While these results provide compelling preclinical evidence, translation to humans requires further validation through studies employing human skin models, long-term supplementation regimens, and clinical trials.

Given its multifunctional role in maintaining skin barrier integrity, ECM stability, and antioxidant defense, NMN holds strong potential as a functional food ingredient and cosmeceutical active ingredient for the prevention and treatment of photoaging. Future studies should also explore synergistic effects with other NAD^+^ precursors or antioxidants, as well as the relative efficacy of oral versus topical administration, to better establish NMN’s therapeutic utility in skin health and anti-aging applications.

## Figures and Tables

**Table 1:** List of primers used for RT-qPCR.

Gene	Assession No.	Primer Sequence
*IL-6*	NM_001314054.1	sense: 5′-GAGGATACCACT CCCAACAG-3′
		anti-sense: 5′-AAGTGCATCATCGTTGTT CA-3′
*TNF-α*	NM_013693.3	sense: 5′-GCCTCTTCTCATTCCTGCTTG-3′
		anti-sense: 5′-CTGATGAGAGGGAGGCCATT-3′
*MMP-1*	NM_032006.3	sense: 5′-TTGCCCAGAGAAAAGCTTCAG-3′
		anti-sense: 5′-TAGCAGCCCAGAGAAGCAACA-3′
*HAS-1*	NM_010441.3	sense: 5′-CTATGCTACCAAGTATACCTCG-3′
		anti-sense: 5′-TCTCGGAAGTAAGATTTGGAC-3′
*HAS-2*	NM_008224.4	sense: 5′-CGGTCGTCTCAAATTCATCTG-3′
		anti-sense: 5′-ACAATGCATCTTGTTCAGCTC-3′
*GAPDH*	NM_008084.3	sense: 5′-GAGGATACCACTCCCAACAG-3′
		anti-sense: 5′-AAGTGCATCATCGTTGTTGTTCA-3′

## Data Availability

The data that support the findings of this study are available on request from the corresponding author.

## Supplementary Materials

The following supporting information can be downloaded at: https://www.mdpi.com/article/10.3390/antiox14121424/s1, Figure S1: Dorsal skin images of SKH-1 hairless mice following UV-B irradiation and NMN or collagen treatments; Table S1: Summary of Week 0 and 10 Skin and Physiological Parameters; Table S2: Quantitative Analysis of Epidermal Thickness and Collagen Intensity in UV-B–Exposed SKH-1 Mouse Skin; Table S3: Quantitative Analysis of Protein Expression Levels of MAPK Pathway in UV-B–Exposed SKH-1 Mouse Skin; Table S4: Quantitative Analysis of mRNA Expression Levels of Inflammatory Cytokines, MMP-1, and Hyaluronan Synthases in UV-B–Exposed SKH-1 Mouse Skin; Table S5: Quantitative Analysis of SOD activity in UV-B–Exposed SKH-1 Mouse serum.
