# Supplementary material for: β-Nicotinamide Mononucleotide Enhances Skin Barrier Function and Attenuates UV-B-Induced Photoaging in Mice
Source: Antioxidants (Basel). 2025 Nov 27;14(12):1424. doi: 10.3390/antiox14121424 (PMC12729516; doi:10.3390/antiox14121424)
Supplement: Supplementary file 1 [file antioxidants-14-01424-s001.zip › antioxidants-3975214-supplementary.pdf]

Supplementary Table 1. Summary of Week 0 and 10 Skin and Physiological Parameters

	Parameter	Vehicle	UV-B	UV-B + NMN 100	UV-B + NMN 300	UV-B + Collagen 300
**Week 0**	Body Weight (g)	27.0 ± 0.7	25.4 ± 1.6	25.6 ± 1.3	24.9 ± 0.8	25.8 ± 1.7
	Moisture (%)	16.4 ± 2.1	17.4 ± 1.7	18.0 ± 1.2	18.4 ± 1.3	18.2 ± 1.3
	TEWL (g/h/m²)	14.8 ± 0.8	14.2 ± 1.6	14.4 ± 2.3	14.4 ± 1.1	13.0 ± 1.6
	Skin Thickness (mm)	0.9 ± 0.1	0.9 ± 0.0	0.8 ± 0.1	0.8 ± 0.1	0.9 ± 0.0
	Elasticity (%area)	129.6 ± 3.2	122.2 ± 6.5	124.4 ± 7.5	121.2 ± 2.7	120.8 ± 4.3
	Skin Wrinkle (mm)	63.0 ± 3.3	61.5 ± 1.9	60.6 ± 2.5	61.3 ± 1.7	59.7 ± 1.7
	Skin Roughness (mm)	62.4 ± 1.9	61.1 ± 2.2	60.1 ± 2.0	59.2 ± 3.6	59.0 ± 1.8
**Week 10**	Body Weight (g)	30.0 ± 1.2	30.1 ± 1.4	30.3 ± 1.3	29.4 ± 1.2	29.5 ± 1.5
	Moisture (%)	16.0 ± 1.0	9.6 ± 1.1	13.4 ± 1.1	15.0 ± 1.2	15.6 ± 1.1
	TEWL (g/h/m²)	17.6 ± 2.7	33.0 ± 1.9	19.8 ± 1.9	23.6 ± 1.1	21.0 ± 2.0
	Skin Thickness (mm)	1.1 ± 0.2	1.8 ± 0.1	1.4 ± 0.1	1.2 ± 0.1	1.2 ± 0.1
	Elasticity (%area)	142.4 ± 4.6	66.2 ± 6.0	103.4 ± 2.7	110.4 ± 3.8	112.4 ± 3.2
	Skin Wrinkle (mm)	63.4 ± 5.2	129.0 ± 3.2	104.7 ± 7.3	88.8 ± 4.7	84.4 ± 9.0
	Skin Roughness (mm)	62.6 ± 2.3	129.6 ± 2.6	101.5 ± 5.9	81.0 ± 3.2	73.4 ± 11.2

Supplementary Table 2. Quantitative Analysis of Epidermal Thickness and Collagen Intensity in UV-B–Exposed SKH-1 Mouse Skin.

Parameter	Vehicle	UV-B	UV-B + NMN 100	UV-B + NMN 300	UV-B + Collagen 300
Epidermal Thickness	5.2 ± 0.8	11.4 ± 1.0	10.5 ± 0.7	6.6 ± 1.1	5.4 ± 0.7
Collagen Intensity	6801.6 ± 185.0	3586.8 ± 239.9	4124.2 ± 523.5	5672.0 ± 178.4	5868.6 ± 60.9

Supplementary Table 3. Quantitative Analysis of Protein Expression Levels of MAPK Pathway in UV-B–Exposed SKH-1 Mouse Skin.

Parameter	Vehicle	UV-B	UV-B + NMN 100	UV-B + NMN 300	UV-B + Collagen 300
p-ERK/ERK (ratio)	1.0 ± 0.3	2.4 ± 0.9	1.3 ± 1.0	0.7 ± 0.5	0.7 ± 0.4
p-JNK/JNK (ratio)	1.0 ± 0.3	2.4 ± 0.4	2.5 ± 0.8	0.8 ± 0.4	0.9 ± 0.4
p-p38/p38 (ratio)	1.0 ± 0.4	2.0 ± 0.2	1.4 ± 0.3	0.9 ± 0.3	0.5 ± 0.2

Supplementary Table 4. Quantitative Analysis of mRNA Expression Levels of Inflammatory Cytokines, MMP-1, and Hyaluronan Synthases in UV-B–Exposed SKH-1 Mouse Skin.

Parameter	Vehicle	UV-B	UV-B + NMN 100	UV-B + NMN 300	UV-B + Collagen 300
TNF-α (fold)	1.0 ± 0.1	17.50 ± 7.83	2.58 ± 1.25	2.67 ± 0.67	1.17 ± 0.83
IL-6 (fold)	1.0 ± 0.1	9.36 ± 8.73	1.27 ± 1.73	0.55 ± 0.64	2.09 ± 4.27
MMP-1 (fold)	1.0 ± 0.3	10.78 ± 7.89	8.89 ± 5.67	6.22 ± 4.00	4.11 ± 2.44
HAS-1 (fold)	1.0 ± 0.1	0.46 ± 0.55	0.82 ± 1.00	0.91 ± 0.55	0.82 ± 0.18
HAS-2 (fold)	1.0 ± 0.2	0.67 ± 0.11	0.33 ± 0.11	1.56 ± 0.44	1.56 ± 0.89

Supplementary Table 5. Quantitative Analysis of SOD activity in UV-B–Exposed SKH-1 Mouse serum.

Parameter	Vehicle	UV-B	UV-B + NMN 100	UV-B + NMN 300	UV-B + Collagen 300
SOD Activity (%)	32.5 ± 10.6	6.6 ± 3.0	29.6 ± 8.5	31.7 ± 9.6	29.9 ± 11.6

(A)

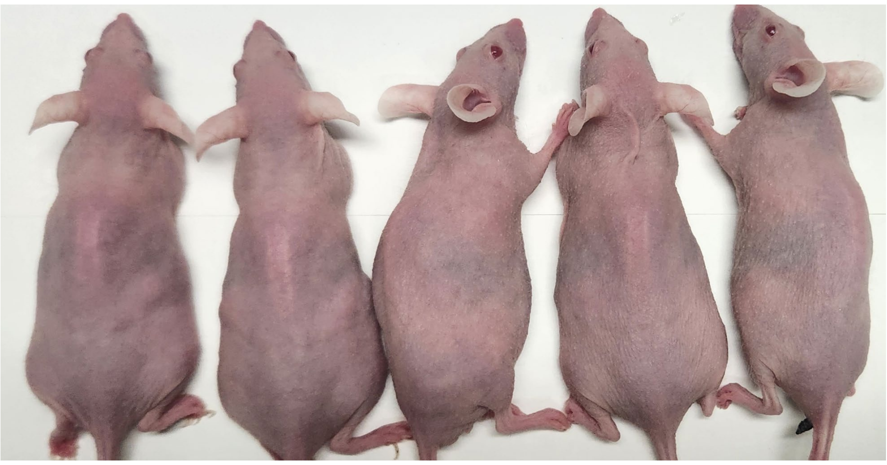

(B)

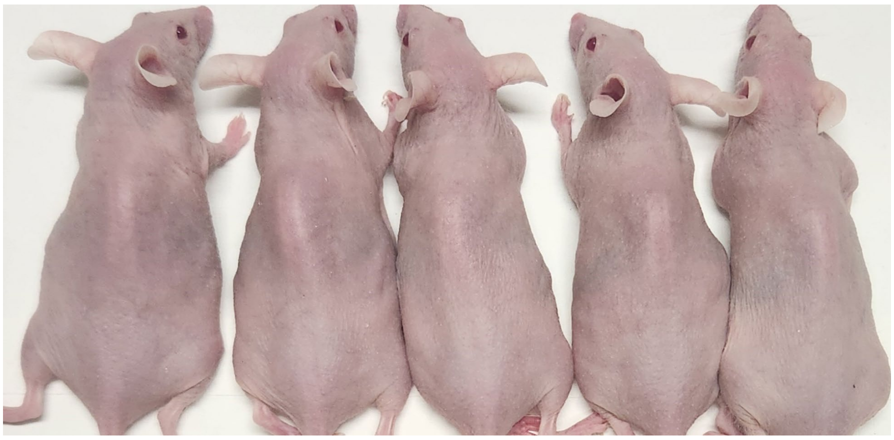

(C)

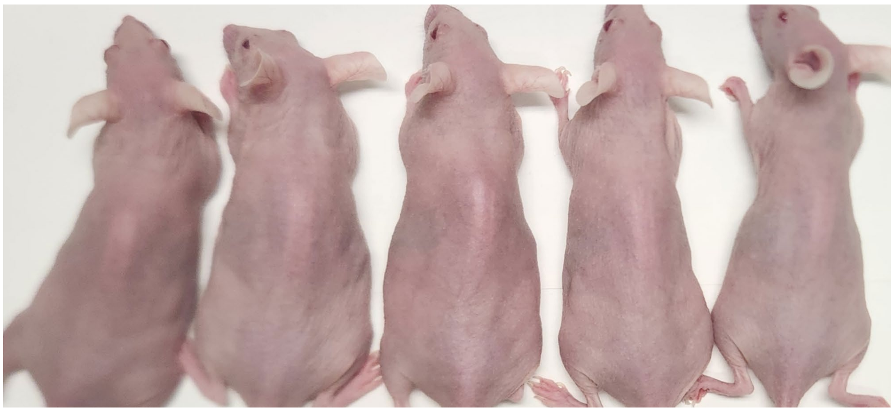

(D)

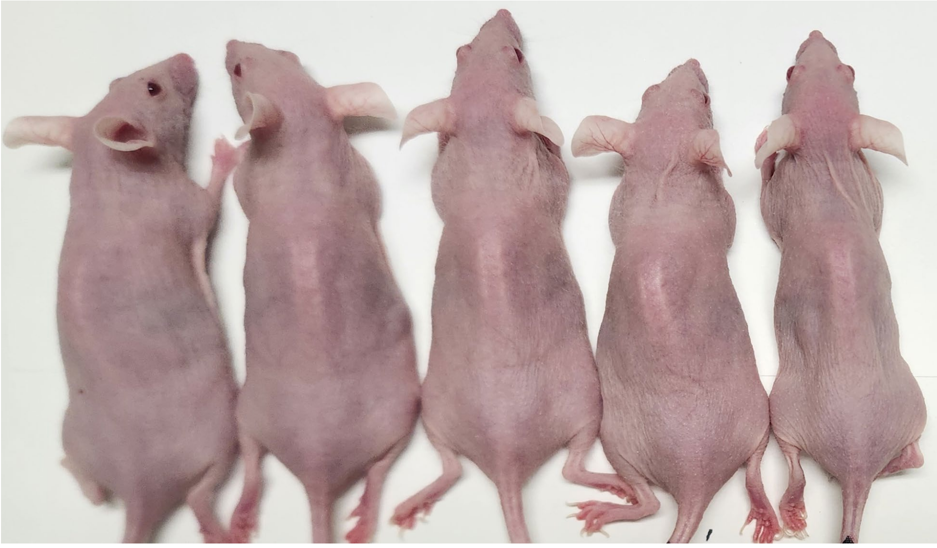

(E)

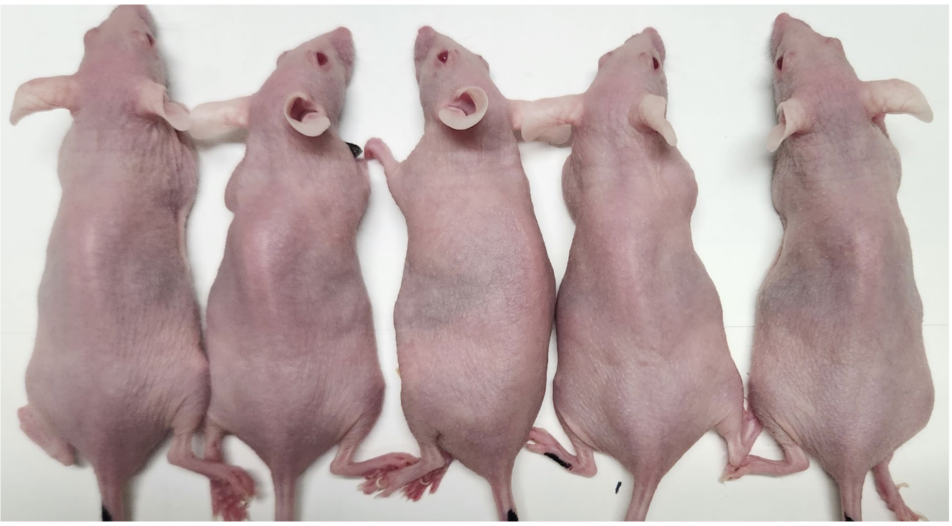

Supplementary Figure 1. Dorsal skin images of SKH-1 hairless mice following UV-B irradiation and NMN or collagen treatments. (A) Vehicle; (B) UVB; (C) UVB+NMN 100; (D) UVB+NMN 300; (E) UVB+Collagen 300
